# Analyzing the Mobile “Digital Divide”: Changing Determinants of Household Phone Ownership Over Time in Rural Bangladesh

**DOI:** 10.2196/mhealth.3663

**Published:** 2015-02-25

**Authors:** Michael Clifton Tran, Alain Bernard Labrique, Sucheta Mehra, Hasmot Ali, Saijuddin Shaikh, Maithilee Mitra, Parul Christian, Keith West Jr

**Affiliations:** ^1^Johns Hopkins Bloomberg School of Public HealthBaltimore, MDUnited States; ^2^The JiVitA Maternal and Child Health ProjectPoschim ParaBangladesh

**Keywords:** digital, mobile Health (mHealth), finances, mobile, phones, Bangladesh, family characteristics, Demography, ownership, socioeconomic factors

## Abstract

**Background:**

We had a unique opportunity to examine demographic determinants of household mobile phone ownership in rural Bangladesh using socioeconomic data collected as part of a multiyear longitudinal cohort study of married women of reproductive age.

**Objectives:**

This paper explores how the demographics of household mobile phone owners have changed over time in a representative population of rural Bangladesh.

**Methods:**

We present data collected between 2008 and 2011 on household mobile phone ownership and related characteristics including age, literacy, education, employment, electricity access, and household wealth among 35,306 individuals. Respondents were enrolled when found to be newly pregnant and contributed socioeconomic information once over the course of the time period serving as a “sample” of families within the population at that time. Univariate and multiple logistic regressions analyses were performed to identify the socioeconomic determinants of household phone ownership.

**Results:**

Across 3 fiscal years, we found that reported household ownership of at least 1 working mobile phone grew from 29.85% in the first fiscal year to 56.07% in the third fiscal year. Illiteracy, unavailability of electricity, and low quartiles of wealth were identified as overall demographic constraints to mobile phone ownership. However, over time, these barriers became less evident and equity gaps among demographic status began to dissipate as access to mobile technology became more democratized. We saw a high growth rate in ownership among households in lower economic standing (illiterate, without electricity, low and lowest wealth index), likely a result of competitive pricing and innovative service packages that improve access to mobile phones as the mobile phone market matures. In contrast, as market saturation is rapidly attained in the most privileged demographics (literate, secondary schooling, electricity, high wealth index), members of the lower wealth quartiles seem to be following suit, with more of an exponential growth.

**Conclusions:**

Upward trends in household mobile phone ownership in vulnerable populations over time underline the potential to leverage this increasingly ubiquitous infrastructure to extend health and finance services across social and economic strata.

## Introduction

The rapid adoption of mobile phones is changing the way individuals communicate on a global scale. Cheap, efficient, and easy-to-use mobile technology has surpassed fixed-lined networks as the primary form of communication in many developing countries. This leapfrogging of landline infrastructure was documented in most of sub-Saharan Africa by 2000 and in Asia by 2002 [1]. While it took landlines 128 years to reach 1 billion users globally, mobile network subscriptions reached 5.9 billion by the end of 2011 with mobile networks doubling in size every 2 years since 2002 [2]. The International Telecommunications Union (ITU) predicts that by 2014, mobile penetration rates will reach 96% worldwide, 100% in developed countries, and 89% in developing countries, with almost an equal number of mobile connections as human beings on the planet [1]. As a prominent driver of mobile growth, developing countries accounted for more than 80% of the 660 million new mobile cellular subscriptions added in 2011 [1]. The remarkable rise in mobile phone uptake supports the proposition that mobile telephony has leapfrogged traditional landline infrastructure to become the preferred platform for communication.

The potential to leverage mobile phones for economic growth has also increased as access to information and communication channels becomes increasingly ubiquitous in developing countries. Economic advantages engendered by mobile technology are multifaceted ranging from providing those with access the ability to search for employment opportunities, negotiate product sales, report emergencies, and reap health and finance services all while reducing associated travel costs [3]. Furthermore, mobile technology serves as a medium to essentially overcome geographic constraints, improve communication, and limit asymmetrical information, characteristic of traditional mechanisms that often require personal travel or reliance on radio, television, and print material [4].

As mobile phones become inextricably linked with development strategies to improve health or provide economic opportunities, inequities of access may prevent the ability to reach segments of the population at the “base of the pyramid”—those most in need of the public health or economic interventions being delivered. Therefore, formulating strategies to maximize access to mobile phones requires an understanding of the changing factors that either enable or limit likelihood of ownership. The concept of differential mobile phone ownership as a result of social, cultural, and economic indicators is referred to as the *digital divide*, which highlights the inequity in access to technologies and subsequent technical services [5-7]*.*

Asia’s rapid mobile adoption has contributed a significant portion of the global market growth and underlines the importance of understanding drivers of mobile phone ownership in countries such as Bangladesh where mobile penetration rates are increasing but the pace of economic development remains slow. Although considered one of the least developed countries in the world according to the United Nations Department of Economic and Social Affairs (UNDESA), in 1993, Bangladesh became the first South Asian country to adopt cellular technology [8,9].

With the support of its parent company, Grameen Bank, Grameen Telecommunication helped spearhead the development of a telecom industry that is now one of the fastest growing industries and largest provider in the last decade [3]. The Bangladesh Telecommunications Regulatory Commission estimated coverage (ie, access to a mobile signal) for 97% of the population, whereas mobile cellular subscribers comprised 97.2% of total subscribers [10]. The total economic impact of the mobile communications sector aggregated from supply-side, demand-side, and intangible benefits across the mobile value chain translated to 2.1% of the gross domestic product in 2004 and increased to 6.2% in 2007 [11]. In 2009, the United Nations Economic and Social Commission for Asia and the Pacific (UNESCAP) conducted a survey in Bangladesh that reported 0.2 mobile cellular subscriptions per 100 population in 2000 grew to 63.8 per 100 population in 2012 with a 20.1% annual growth rate (Table 1) [12]. Table 1 shows the variability of mobile phone diffusion throughout markets in Asia and the Pacific. In addition to income, the differences in adoption suggest competing explanations of penetration drivers such as demographic determinants [13].

**Table 1:** Asia and the pacific mobile cellular subscriptions in 2000, 2008, and 2012.^a^

Country	Mobile cellular subscriptions			
	Per 100 population			% change per annum 2000-2012
	2000	2008	2012	
Japan	53.1	87.2	109.4	5.8
Cambodia	1.0	30.7	132.0	44.0
Kyrgyzstan	0.2	65.2	124.8	17.6
Bangladesh	0.2	30.7	63.8	20.1
Papua New Guinea	0.2	13.3	37.8	29.7
Asia and the Pacific	6.5	50.9	85.6	13.9
World	12.1	59.9	89.5	10.5

^a^ Source: data extracted from UNESCAP statistics division [12].

At its simplest, basic-entry level, mobile phone ownership provides an ability to strengthen communication using voice and text messages. Bridging the mobile digital divide among economic and social groups such as the poor and women, respectively, could spur economic development and reconcile the inequitable distribution of power that stems from differential access to technology [5,11]. With such promise, socioeconomic groups within parts of rural Bangladesh where mobile phone ownership remains low is of particular concern because these populations lack the ability to leverage the maximum potential of access to information and resources through mobile communications. Often, it is membership in these lower socioeconomic status (SES) subgroups that also bear the greatest burden of ill health [14].

This paper explores how the demographics of household mobile phone owners have changed over time in a representative population of rural Bangladesh. Understanding the demographics of mobile phone ownership is essential to conceptualize, organize, and implement interventions that target vulnerable populations; if household ownership is crucial to program effectiveness, the strategy may have to provide phones or work on modifying the factors that influence ownership. Programs that target individuals, such as those offering customized reminders or information-providing messages, may require personal or household ownership of a phone for maximum impact, although there is little evidence to suggest this is true [15].

## Methods

### Overview

In 2008, the United Nations deemed household mobile phone ownership the best indication of adoption because household mobile phones are usually accessible to every member in the house. However, it is important to note that this assumption may not hold true in conservative communities where women’s access to household assets is restricted or controlled by a patriarch [16]. Additionally, measuring household mobile phone ownership protects from identifying endogenous factors associated with multiple SIM card subscriptions per individual [2].

### Recruitment

In seeking a representative population site resonant with populations across the greater Gangetic floodplain, Gaibandha and Rangpur districts were selected based on maternal health reports, remoteness, and rural quality (eg, mostly villages linked by unpaved roads, surrounded by rice fields) [17]. The defined research area is approximately 435 km^2^ in size with a population density of approximately 1000 per km^2^ and mainly agrarian in nature (eg, seasonality and crop mix, weekly market network) [18].

Data from JiVitA-3, a randomized controlled trial (RCT) conducted in rural northwestern Bangladesh from 2008 to 2011 [19] was used for this analysis. During this trial aiming to assess the effect of nutrient supplementation on infant mortality, 44,467 pregnant women were enrolled from a cohort of approximately 120,000 married women of reproductive age [20]. A socioeconomic assessment was conducted on all consenting newly pregnant women enrolled in the RCT and household mobile phone ownership was 1 characteristic. The aim of this analysis was to model the predictors of household phone ownership over time in a rural setting of Bangladesh where population, health, agriculture, and infrastructure broadly reflect the national rural population [17].

### Statistical Analyses

The variables included in the analysis were women’s age, parity (number of children), literacy (measured as the reported ability to read and write a letter in the Bengali language), education, employment, access to electricity, and SES. Age was categorized as ≤19, 20-24, 25-29, and ≥30 years; parity as 0, 1-3, and >3; and education as none, primary (class 1-9), and secondary schooling (class 10 and above). Age and parity were specific to the married female respondent, whereas level of education, literacy, and employment information were reported personally by the respondent and on behalf of her husband, providing information on both members of the household. Household access to electricity, employment of husband and wife, and literacy were categorized as dichotomous variables. As the outcome variable, mobile phone ownership was dichotomous, classified as either no household mobile phone ownership or ≥1 household mobile phones.

Exploratory data analysis was conducted to determine relevant variables. Each respective variable was incorporated into a univariate analysis to identify potential determinants of mobile phone ownership followed by a multiple logistic regression analysis to adjust for confounding and identify the significant predictors of mobile phone ownership. Univariate and multiple logistic regression analyses assessed associations between household mobile phone ownership and demographics. Variation inflation factor (VIF) was used to check for collinearity between education, employment, literacy, and SES. Outliers were accounted for using DFFITS analysis. Listwise deletion was used for a complete case analysis to omit missing data. Lastly, a test for homogeneity was used to assess effect modification of the association between literacy, education, occupation, electricity, and wealth index (WI) with mobile phone ownership. An a priori level of statistical significance was set at *P*<.05. All of the values were unique; that is, individuals were not followed longitudinally. STATA version 12.0 (StataCorp LP, College Station, TX, USA) was used for statistical analyses.

To evaluate temporal trends, the demographics of household mobile phone ownership were measured over a 3-year fiscal period that began in July and ended in June from 2008 to 2011. In doing so, the first 6 months (January 2008-June 2008) of the dataset were excluded to capture the latest entries (July 2011) in the last fiscal year. For this period, data on 35,306 trial participants were used for the analysis and are presented here. Once stratified by fiscal year, univariate and multivariate analyses were used to assess whether demographic determinants of phone ownership changed over time.

### Socioeconomic Status

A principal component analysis was previously used to construct a WI, which factored durable assets, dwelling characteristics, productive assets, and land ownership [21,22]. These indexes are representative of SES because they are more easily and reliably reported than income or consumption expenditure data in developing countries [21]. As in Filmer et al [23], WI was categorized into ordinal variables categorizing it into quartiles (lowest, low, high, and highest) of SES.

## Results

**Figure 1:**
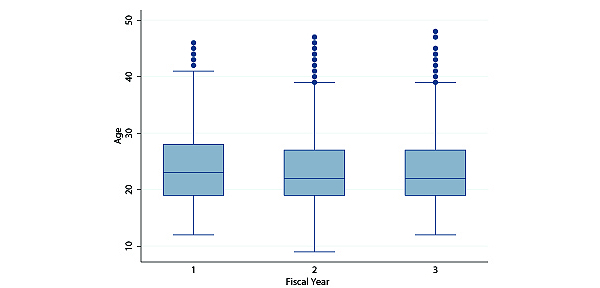
Box plot of respondents’ ages who owned mobile phones by fiscal year.

Overall, the youngest respondent with a household mobile phone was aged 9 years, whereas the oldest respondent was aged 48 years. The median age of respondents who owned a household mobile phone in fiscal year 1 was 23 years (IQR 9), whereas the median age for fiscal years 2 and 3 was 22 years (IQR 8) (Figure 1). Table 2 shows the association between demographic characteristics and non–phone ownership; Table 3 shows the association between demographic characteristics and phone ownership stratified over 3 fiscal years. Overall, phone ownership increased by fiscal year (year 1: 29.85%, 4178/13,996; year 2: 39.91%, 4842/12,132; year 3: 56.07%, 5107/9109).

**Figure 2:**
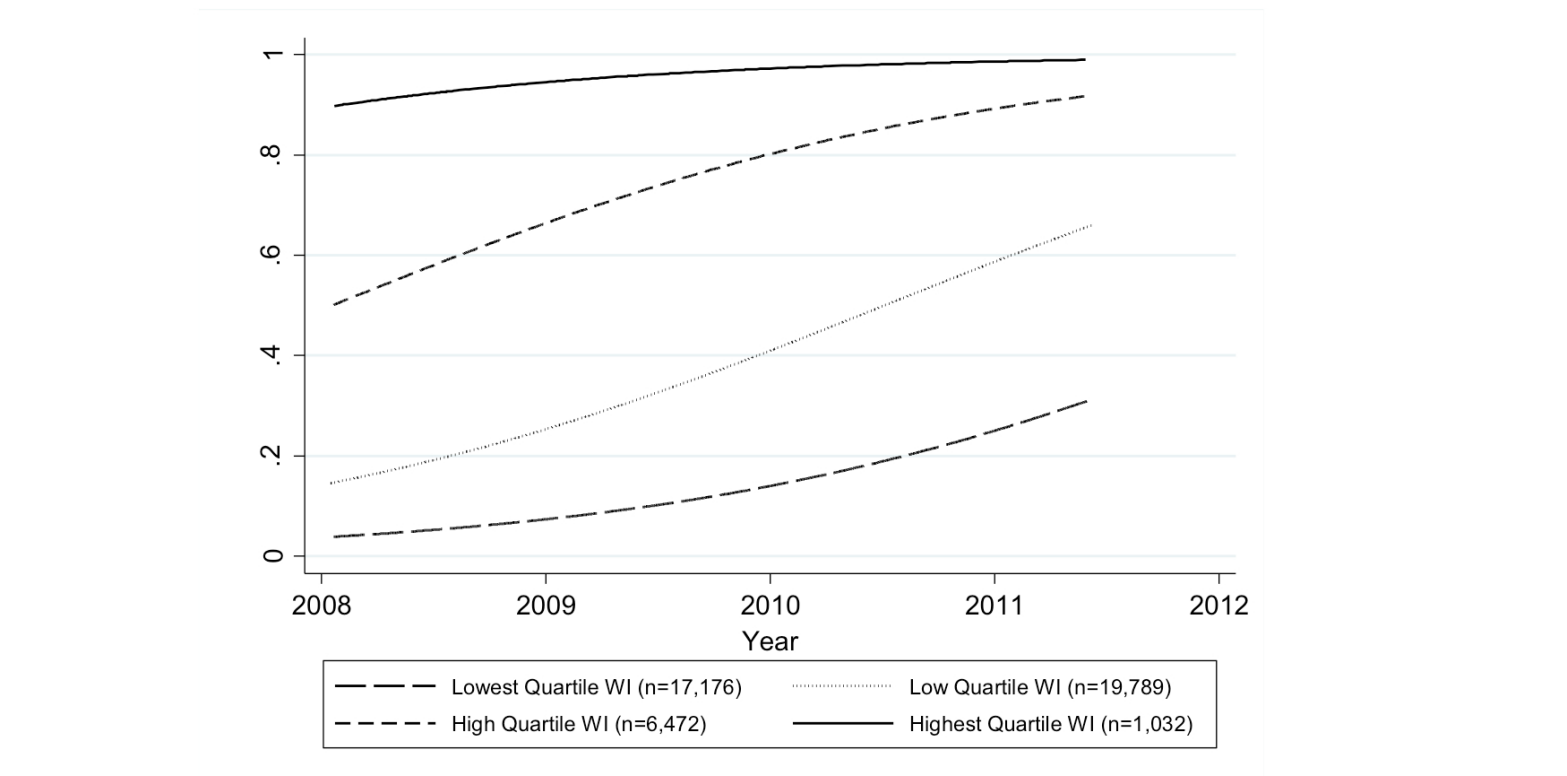
Trends (*P*<.001) in household mobile phone ownership by wealth index (WI), 2008-2011.

The proportion of households in the low socioeconomic groups owning a mobile phone changed dramatically over time. Figure 2 shows that when stratified by WI, 91.6% of households in the lowest quartile did not own a mobile phone, whereas only 3.9% of households in the highest quartile did not own a mobile phone in fiscal year 1 (Figure 2). However, by fiscal year 3, 70.5% of households in the lowest quartile and only 1.9% of households in the highest quartile did not own a mobile phone.

Tables 4-6 show the unadjusted and adjusted odds ratios of mobile phone ownership for all demographic variables across fiscal years 1, 2, and 3, respectively. Unadjusted univariate analysis shows that the older the wives (respondents) or the more children in a household, the less likely it was to own a mobile phone because it was negatively associated and continued to decrease across fiscal years 1 and 3. Wife’s employment (fiscal year 1: OR 0.74, 95% CI 0.69-0.79, *P*<.001; fiscal year 3: OR 0.78, 95% CI 0.71-0.84, *P*<.001) was the only variable that had an overall increase in the odds of owning a mobile phone over time but remained negatively associated. Wife’s literacy, husband’s literacy, wife’s education, husband’s education, electricity, and WI were all positively associated with mobile phone ownership that also attenuated over time. There was a decreasing dose-response relationship over the 3-year fiscal periods for the respondents aged 25-29 and ≥30 years, all parity groups (1-3 and ≥4), wife’s literacy, husband’s literacy, wife’s education, husband’s education, and for the low and high quartiles of WI.

**Table 2:** Demographic characteristics of mobile phone nonowners by fiscal year, 2009-2011.

Demographic characteristics			July 2008 to June 2009 (n=13,996)	July 2009 to June 2010 (n=12,132)	July 2010 to June 2011 (n=9109)
**Households, n (%)**					
	Non–mobile phone owners		9818 (70.15)	7290 (60.09)	4002 (43.93)
**Wife’s age (years)**					
	Mean (SD)		24.41 (6.5)	24.67 (6.5)	24.91 (6.5)
	**Range, n (%)**				
		≤19	2694 (69.20)	1874 (55.10)	993 (37.70)
		20-24	2827 (68.63)	2090 (58.76)	1054 (41.00)
		25-29	2103 (69.61)	1694 (63.19)	987 (48.31)
		≥30	2194 (74.05)	1632 (65.46)	968 (52.02)
**Parity, n (%)**					
	0		2470 (62.36)	1866 (49.69)	1082 (33.59)
	1-3		6141 (71.85)	4605 (63.35)	2505 (48.09)
	≥4		1207 (81.12)	819 (73.92)	415 (61.12)
**Wife literate, n (%)**					
	No		5205 (85.79)	3761 (77.21)	2054 (62.00)
	Yes		4613 (58.19)	3526 (48.58)	1947 (33.60)
**Husband literate, n (%)**					
	No		5724 (86.96)	4307 (77.72)	2442 (63.49)
	Yes		4091 (55.22)	2970 (45.18)	1555 (29.61)
**Wife’s education, n (%)**					
	No schooling		3872 (86.29)	2759 (78.49)	1532 (63.62)
	Primary (1-9)		5783 (67.84)	4415 (57.15)	2412 (41.06)
	Secondary (≥10)		163 (16.57)	116 (13.00)	58 (7.02)
**Husband’s education, n (%)**					
	No schooling		5361 (87.71)	3956 (78.38)	2230 (63.66)
	Primary (1-9)		3574 (66.44)	2620 (54.75)	1406 (38.01)
	Secondary (≥10)		883 (32.25)	714 (31.04)	366 (19.19)
**Wife employed, n (%)**					
	No		5635 (67.63)	4042 (57.89)	2100 (41.17)
	Yes		4183 (73.85)	3248 (63.07)	1902 (47.46)
**Husband employed, n (%)**					
	No		13 (56.52)	4 (57.14)	3 (60.00)
	Yes		9805 (70.17)	7286 (60.09)	3999 (43.93)
**Electricity, n (%)**					
	No		8612 (76.79)	6364 (67.50)	3465 (50.47)
	Yes		1205 (43.36)	925 (34.22)	537 (23.93)
**Wealth index (WI),** **n (%)**					
	Lowest quartile		5081 (91.58)	3896 (84.02)	2155 (70.54)
	Low quartile		4156 (68.31)	3066 (56.28)	1699 (39.80)
	High quartile		568 (27.97)	323 (18.26)	143 (9.40)
	Highest quartile		13 (3.90)	5 (1.80)	5 (1.89)

**Table 3:** Demographic characteristics of mobile phone owners by fiscal year, 2009-2011.

Demographic characteristics			July 2008 to June 2009 (n=13,996)	July 2009 to June 2010 (n=12,132)	July 2010 to June 2011 (n=9109)
**Households, n (%)**					
	Mobile owners		4178 (29.85)	4842 (39.91)	5107 (56.07)
**Wife’s age (years)**					
	Mean (SD)		23.96 (6.3)	23.64 (6.3)	23.56 (6.2)
	**Range, n (%)**				
		≤19	1199 (30.80)	1527 (44.90)	1641 (62.30)
		20-24	1292 (31.37)	1467 (41.24)	1517 (59.00)
		25-29	918 (30.39)	987 (36.81)	1056 (51.69)
		≥30	769 (25.95)	861 (34.54)	893 (47.98)
**Parity, n (%)**					
	0		1491 (37.64)	1889 (50.31)	2139 (66.41)
	1-3		2406 (28.15)	2664 (36.65)	2704 (51.91)
	≥4		281 (18.88)	289 (26.08)	264 (38.88)
**Wife literate, n (%)**					
	No		862 (14.21)	1110 (22.79)	1259 (38.00)
	Yes		3315 (41.81)	3732 (51.42)	3848 (66.40)
**Husband literate, n (%)**					
	No		858 (13.04)	1235 (22.28)	1404 (36.51)
	Yes		3318 (44.78)	3604 (54.82)	3696 (70.39)
**Wife’s education, n (%)**					
	No schooling		615 (13.71)	756 (21.51)	876 (36.38)
	Primary (1-9)		2742 (32.16)	3310 (42.85)	3463 (58.94)
	Secondary (≥10)		821 (83.43)	776 (87.00)	768 (92.98)
**Husband’s education, n (%)**					
	No schooling		751 (12.29)	1091 (21.62)	1273 (36.34)
	Primary (1-9)		1805 (33.56)	2165 (45.25)	2293 (61.99)
	Secondary (≥10)		1622 (64.75)	1586 (68.96)	1541 (80.81)
**Wife employed, n (%)**					
	No		2697 (32.37)	2940 (42.11)	3001 (58.83)
	Yes		1481 (26.15)	1902 (36.91)	2106 (52.54)
**Husband employed, n (%)**					
	No		10 (43.48)	3 (42.86)	2 (40.00)
	Yes		4168 (29.83)	4839 (39.91)	5105 (56.07)
**Electricity, n (%)**					
	No		2603 (23.21)	3064 (32.50)	3400 (49.53)
	Yes		1574 (56.64)	1778 (65.78)	1707 (76.07)
**Wealth index (WI),** **n (%)**					
	Lowest quartile		467 (8.42)	741 (15.98)	900 (29.46)
	Low quartile		1928 (31.69)	2382 (43.72)	2570 (60.20)
	High quartile		1463 (72.03)	1446 (81.74)	1378 (90.60)
	Highest quartile		320 (96.10)	273 (98.20)	259 (98.11)

**Table 4:** Unadjusted and adjusted odds ratio (OR) and 95% confidence intervals (95% CI) of reporting household mobile phone ownership by demographics for fiscal year 1 (n=13,996), July 2008-June 2009.

Demographic characteristics		Unadjusted		Adjusted	
		OR (95% CI)	*P*	OR (95% CI)	*P*
**Wife’s age (years)**					
	≤19	1.00		1.00	
	20-24	1.03 (0.93-1.13)	.58	1.25 (1.09-1.43)	.002
	25-29	0.98 (0.89-1.09)	.71	1.23 (1.04-1.45)	.02
	≥30	0.79 (0.71-0.88)	<.001	1.15 (0.96-1.39)	.14
**Parity**					
	0	1.00		1.00	
	1-3	0.65 (0.59-0.70)	<.001	0.80 (0.70-0.92)	.002
	≥4	0.39 (0.33-0.45)	<.001	0.72 (0.58-0.89)	.004
**Wife literate**					
	No	1.00		1.00	
	Yes	4.34 (3.99-4.72)	<.001	1.63 (1.39-1.91)	<.001
**Husband literate**					
	No	1.00		1.00	
	Yes	5.41 (4.97-5.89)	<.001	1.58 (1.33-1.88)	<.001
**Wife’s education**					
	No schooling	1.00		1.00	
	Primary (1-9)	2.99 (2.71-3.29)	<.001	0.87 (0.73-1.03)	11
	Secondary (≥10)	31.71 (26.27-38.29)	<.001	1.84 (1.33-1.88)	<.001
**Husband’s education**					
	No schooling	1.00		1.00	
	Primary (1-9)	3.61 (3.28-3.97)	<.001	1.34 (1.12-1.60)	.002
	Secondary (≥10)	13.11 (11.72-14.67)	<.001	2.17 (1.77-2.68)	<.001
**Wife employed**					
	No	1.00		1.00	
	Yes	0.74 (0.69-0.79)	<.001	0.75 (0.68-0.82)	<.001
**Husband employed**					
	No	1.00		1.00	
	Yes	0.55 (0.24-1.26)	.16	1.16 (0.39-3.45)	.78
**Electricity**					
	No	1.00		1.00	
	Yes	4.32 (3.96-4.71)	<.001	1.70 (1.53-1.89)	<.001
**Wealth index (WI)**					
	Lowest quartile	1.00		1.00	
	Low quartile	5.05 (4.53-5.63)	<.001	3.41 (3.04-3.84)	<.001
	High quartile	28.02 (24.47-32.09)	<.001	11.04 (9.47-12.88)	<.001
	Highest quartile	267.81 (152.59-470.06)	<.001	61.73 (34.69-109.84)	<.001

**Table 5:** Unadjusted and adjusted odds ratio (OR) and 95% confidence intervals (95% CI) of reporting household mobile phone ownership by demographics for fiscal year 2 (n=12,132), July 2009-June 2010.

Demographic characteristics		Unadjusted		Adjusted	
		OR (95% CI)	*P*	OR (95% CI)	*P*
**Wife’s age (years)**					
	≤19	1.00		1.00	
	20-24	0.86 (0.78-0.95)	.002	1.06 (0.92-1.21)	.42
	25-29	0.72 (0.65-0.79)	<.001	0.92 (0.78-1.08)	.30
	≥30	0.65 (0.58-0.72)	<.001	0.90 (0.75-1.09)	.29
**Parity**					
	0	1.00		1.00	
	1-3	0.57 (0.53-0.62)	<.001	0.83 (0.73-0.95)	.006
	≥4	0.35 (0.30-0.40)	<.001	0.6 (0.61-0.95)	.02
**Wife literate**					
	No	1.00		1.00	
	Yes	3.59 (3.31-3.89)	<.001	1.37 (1.19-1.58)	<.001
**Husband literate**					
	No	1.00		1.00	
	Yes	4.23 (3.91-4.58)	<.001	1.52 (1.29-1.79)	<.001
**Wife’s education**					
	No schooling	1.00		1.00	
	Primary (1-9)	2.74 (2.49-3.00)	<.001	1.02 (0.87-1.19)	.79
	Secondary (≥10)	24.41 (19.77-30.15)	<.001	2.43 (1.82-3.24)	<.001
**Husband’s education**					
	No schooling	1.00		1.00	
	Primary (1-9)	2.99 (2.74-3.27)	<.001	1.17 (0.99-1.38)	.07
	Secondary (≥10)	8.05 (7.21-8.99)	<.001	1.53 (1.26-1.87)	<.001
**Wife employed**					
	No	1.00		1.00	
	Yes	0.81 (0.75-0.87)	<.001	0.86 (0.79-0.94)	.002
**Husband employed**					
	No	1.00		1.00	
	Yes	0.89 (0.19-3.96)	.87	1.69 (0.27-10.51)	.57
**Electricity**					
	No	1.00		1.00	
	Yes	3.99 (3.65-4.37)	<.001	1.76 (1.58-1.96)	<.001
**Wealth index (WI)** ^a^					
	Lowest quartile	1.00		1.00	
	Low quartile	4.09 (3.71-4.49)	<.001	2.89 (2.61-3.21)	<.001
	High quartile	23.54 (20.38-27.18)	<.001	10.33 (8.79-12.12)	<.001
	Highest quartile	287.07 (118.13-697.66)	<.001	76.97 (31.37-188.85)	<.001

^a^ WI is constructed from a principal component analysis of dwelling characteristics, durable assets, productive assets, and land ownership.

**Table 6:** Unadjusted and adjusted odds ratio (OR) and 95% confidence intervals (95% CI) of reporting household mobile phone ownership by demographics for fiscal year 3 (n=9109), July 2010-June 2011.

Demographic characteristics		Unadjusted		Adjusted	
		OR (95% CI)	*P*	OR (95% CI)	*P*
**Wife’s age (years)**					
	≤19	1.00		1.00	
	20-24	0.87 (0.78-0.97)	.02	1.22 (1.03-1.44)	.02
	25-29	0.65 (0.58-0.73)	<.001	1.03 (0.85-1.26)	.75
	≥30	0.56 (0.49-0.63)	<.001	0.95 (0.77-1.18)	.67
**Parity**					
	0	1.00		1.00	
	1-3	0.55 (0.49-0.59)	<.001	0.79 (0.67-0.93)	.005
	≥4	0.32 (0.27-0.38)	<.001	0.68 (0.68-0.89)	.004
**Wife literate**					
	No	1.00		1.00	
	Yes	3.22 (2.95-3.52)	<.001	1.28 (1.09-1.49)	.002
**Husband literate**					
	No	1.00		1.00	
	Yes	4.13 (3.78-4.52)	<.001	1.85 (1.55-2.21)	<.001
**Wife’s education**					
	No Schooling	1.00		1.00	
	Primary (1-9)	2.51 (2.28-2.77)	<.001	0.99 (0.84-1.18)	.96
	Secondary (≥10)	23.16 (17.51-30.63)	<.001	2.53 (1.76-3.62)	.03
**Husband’s education**					
	No schooling	1.00		1.00	
	Primary (1-9)	2.86 (2.59-3.14)	<.001	0.97 (0.80-1.16)	.70
	Secondary (≥10)	7.38 (6.46-8.43)	<.001	1.27 (1.02-1.58)	.03
**Wife employed**					
	No	1.00		1.00	
	Yes	0.78 (0.71-0.84)	<.001	0.84 (0.76-0.94)	.001
**Husband employed**					
	No	1.00		1.00	
	Yes	1.92 (0.32-11.47)	.48	2.09 (0.19-21.97)	.54
**Electricity**					
	No	1.00		1.00	
	Yes	3.24 (2.91-3.61)	<.001	1.44 (1.27-1.64)	<.001
**Wealth index (WI)** ^a^					
	Lowest quartile	1.00		1.00	
	Low quartile	3.62 (3.28-3.99)	<.001	2.66 (2.38-2.96)	<.001
	High quartile	23.07 (19.10-27.87)	<.001	10.73 (8.73-13.19)	<.001
	Highest quartile	124.03 (51.02-301.54)	<.001	38.66 (15.71-95.15)	<.001

^a^ WI is constructed from a principal component analysis of dwelling characteristics, durable assets, productive assets, and land ownership.

When adjusting for all variables (wife’s age, parity, literacy, education, employment, WI), age, and wives and husbands with just a primary education were no longer significant by fiscal year 3, whereas husband’s employment was not statistically significant across all fiscal years. Multiple logistic regression analysis showed that wife’s employment (fiscal year 1: OR 0.75, 95% CI 0.68-0.82, *P*<.001; fiscal year 3: OR 0.84, 95% CI 0.76-0.94, *P*=.001) and husband’s literacy (fiscal year 1: OR 1.58, 95% CI 1.33-1.88, *P*<.001; fiscal year 3: OR 1.85, 95% CI 1.55-2.21; *P*<.001) were the only 2 demographic variables that had an overall increase from fiscal year 1 to fiscal year 3, whereas all other statistically significant demographic variables had an overall decrease in odds of owning a household mobile phone. Wives with just a primary education were negatively associated with mobile phone ownership despite having an overall increase in odds. Husbands with a secondary education, electricity, and all quartiles of WI (lowest, low, high, and highest) were all positively associated with mobile phone ownership that also attenuated over time. There was a decreasing dose-response relationship for husband’s education (primary and secondary, respectively) and the low quartile WI, whereas there was an increasing dose-response relationship for households where wives had a secondary education.

Low VIF values indicate that collinearity is not present between wealth (1.28), employment (1.03), and education (2.81).

## Discussion

### Principal Findings

Based on global trends, the ITU suggested in 2013 that as market saturation is attained within a given population, growth rates in mobile phone ownership would decrease [1]. In our dataset, household mobile phone ownership among demographic variables changed rapidly over time. Market saturation (>90%) was observed in households where wives had a secondary education (93.0%) or in households within the high (90.6%) and highest (98.1%) quartiles of WI by fiscal year 3. Market-saturated demographic groups can be considered early adopters of mobile phones, in which uptake is fastest. These early adopters rapidly reached saturation because they were able to afford the technologies from the outset when mobile phone costs were relatively high. In contrast, low SES groups such as households in the low (60.2%) and lowest (29.5%) quartiles of WI represent a larger proportion of the total sample size (83.1%), but have a low proportion of ownership. These low SES groups are considered late adopters, in which uptake is slowest.

In examining predictors of ownership by fiscal year, the velocity of change in ownership indicates which demographic factor achieves the fastest rate of growth. Despite a slower trajectory among households of lower economic standing, the growth rate of mobile phone ownership is increasing exponentially as seen in these late adopter groups because the price of “entry” into the mobile marketplace likely declined over time. The change in the proportion of household ownership when stratified by WI shows among the lowest quartile WI there is a sustained exponential growth, where the digital divide is being bridged over time (Figure 2). The statistic of ownership of at least 1 mobile phone across the entire study period (35.8% among the households surveyed) is grossly misleading. When stratified by fiscal year, phone ownership was 29.85% (4178/13,996), 39.91% (4842/12,132), and 56.07% (5107/9109), for fiscal years 1, 2, and 3, respectively.

**Figure 3:**
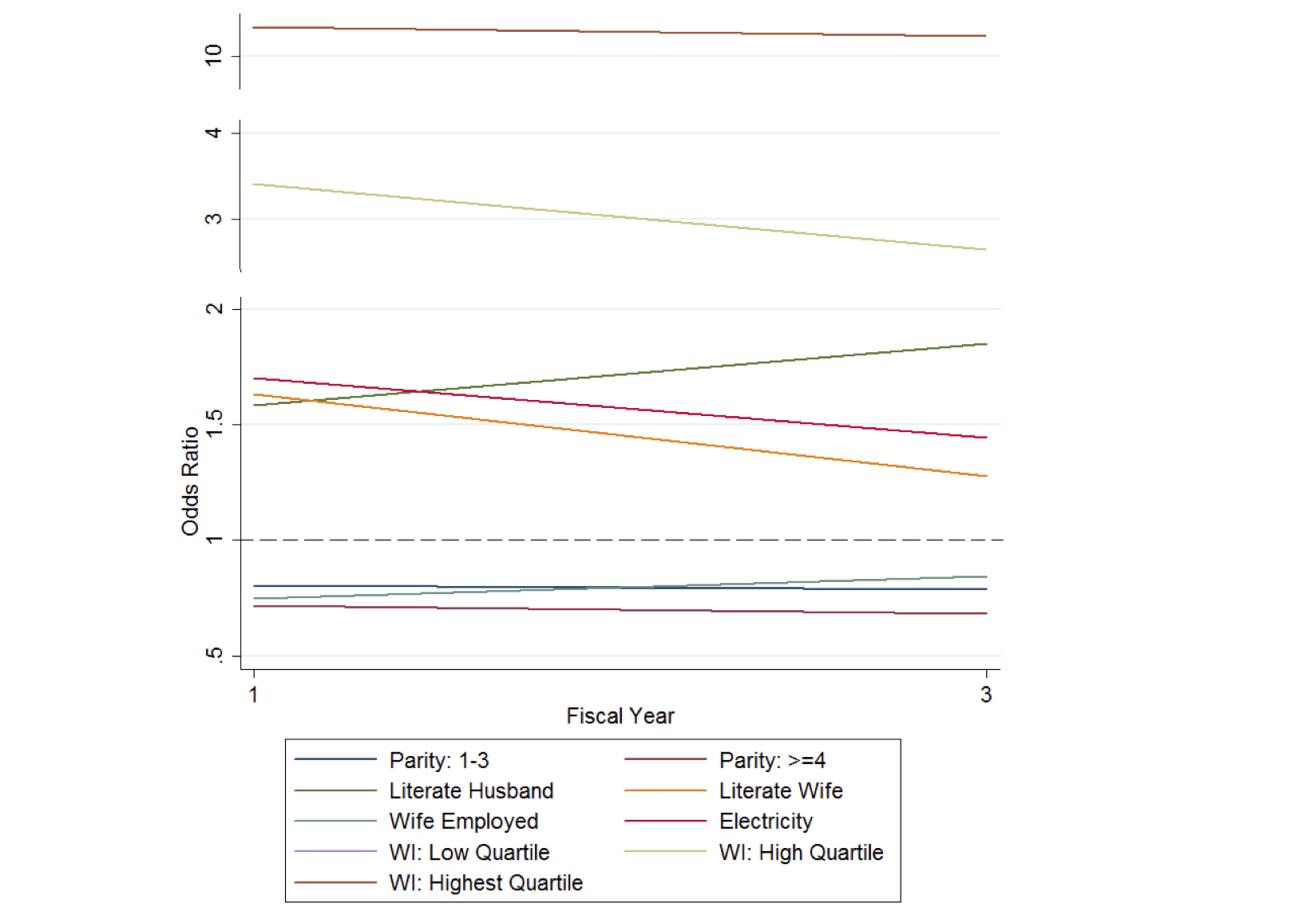
Odds ratio of statistically significant demographic variables (*P*<.05) over fiscal years 1 and 3.

Although overall phone ownership increased by fiscal year, the overwhelming trend in which the odds of owning a mobile phone attenuated over time suggests that the factors that distinguish people from one another—education, electricity, and WI—are important early in the mobile “revolution” when prices are likely high and the technology new (Figure 3). However, as mobile phones become more available and the markets mature, access to mobile technology is more democratized and equity gaps begin to dissipate.

As market saturation is rapidly attained in the most privileged demographics (literate, secondary schooling, electricity, high WI) likely because of a combination of early adoption, willingness-to-pay, and affordability, members of the lower wealth quartiles seem to be following suit with more of an exponential growth mirroring the global trends seen elsewhere [1]. As stated by Grameenphone in their 2010 Annual Report, price competition across network operators led to a plateauing of the annual revenue per unit reported by this major network operator, but this also suggests a decline in prices charged for services given the documented rate of subscriber growth during this period [24]. Our data support that these market forces seem to directly impact the inequity of what began in the early days of mobile introduction in Bangladesh as a marked mobile “digital divide” resulting in a trend of increasing ownership less likely to be driven by markers of SES such as literacy, educational attainment, age, or wealth.

The high growth rates of ownership among the most vulnerable subpopulations in our analysis and the decreasing predictive capacity of variables initially strongly associated with risk of phone ownership suggests that sociodemographic constraints do not represent an insurmountable barrier to mobile phone ownership over the life-course of mobile phone introduction into a population. These data elegantly illustrate how the digital divide is closing over a relatively short span of time, a likely result of changing market characteristics and resulting demographic predictors of ownership over time. Nonetheless, despite these closing gaps, there still remains a population segment that is largely without access to mobile phone ownership. Households in the lowest quartile of WI with more children, where the wife has either a primary education or no schooling despite being employed are the least likely to own a mobile phone. Recognizing and targeting this “base of the pyramid” group is important to alleviate the “inequitable distribution of power that stems from differential access to information and communications technology resources” [5].

### Increasing Equity to Women

The data show that the probability of owning a mobile phone is greatest in households where both the wife and husband are literate. Although there is interaction between ownership and literacy between the wife and husband, the difference in mobile phone ownership among households with discordant couples likely reflects gender-power differences in Bangladesh. In discordant couples, the probability of owning a mobile phone is greater in households in which only the husband is literate compared with households in which only the wife is literate, thereby highlighting a possible interesting proxy of inequity in purchasing power of the women for the household, even when their education level may be higher than their husbands’ education level.

The disparity brings to mind the larger issue of prevailing sociocultural norms practiced in rural Bangladesh. Women are often subject to discrimination engendered by a highly patriarchal social system that determines power relations within households and the bargaining power of household members [25]. In efforts to deconstruct the associated ideologies that precipitate discrimination, it is important to negotiate cultural norms that place value on women’s work and education [25]. Mobile phone ownership and access may be a vehicle through which women can reframe their role in the household or position in society, empowered by connectivity.

**Figure 4:**
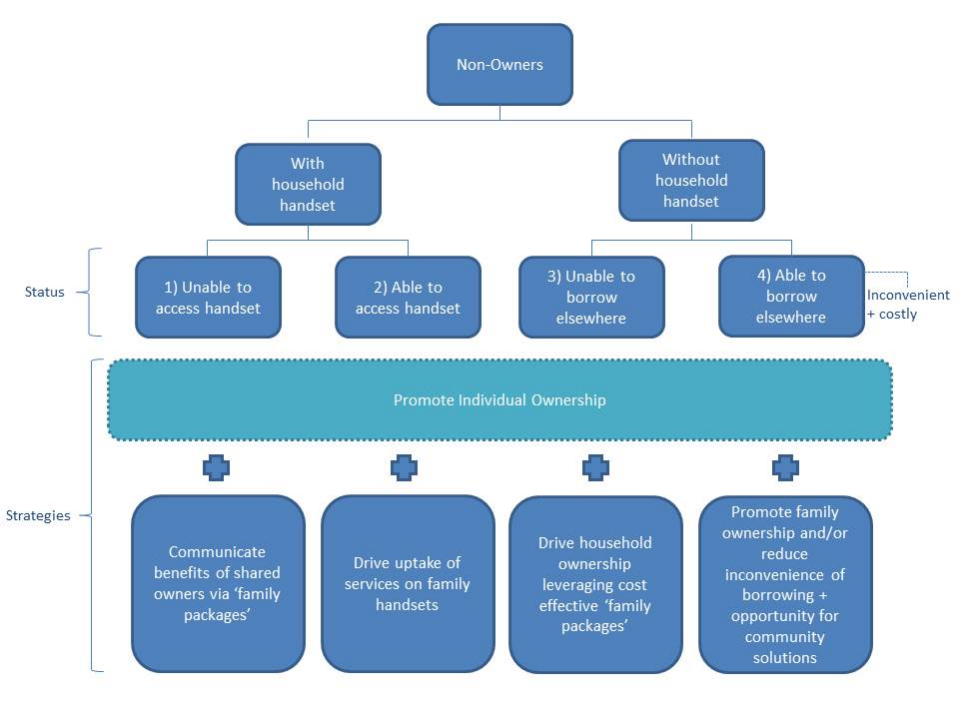
GSMA mWomen strategies to promote individual ownership. Source: [16].

In a 2012 study of mobile phone access among women at the proverbial “base of the pyramid,” researchers identified that, globally, a woman is 21% less likely to own a mobile phone than a man due to social, cultural, and economic reasons [16]. This specific digital divide was described as a gender gap that translated to roughly US $13 billion dollars of associated missed market opportunities [16]. Clearly, the varying social, cultural, and economic drivers of adoption, as described in this analysis, should be targeted by the mobile industry while keeping in mind the struggles of the members of these socioeconomic strata to prioritize food, housing, and health care. In 2014, the Groupe Speciale Mobile Association (GSMA) mWomen Program aims to reduce the inequitable distribution of mobile phones by 50% consequently increasing mobile connectivity to more than 150 million women in emerging markets [16]. In doing so, a number of approaches have been proposed to bridge mobile ownership inequity in an effort to provide access to more members of the lowest socioeconomic strata sooner than current trends might forecast (Figure 4).

As mobile phones continue to penetrate the developing market, economies of scale and scope, specialization and speed all play a factor in the growth of the mobile phone industry and the improvement of connectivity. To harness the full potential of connectivity using mobile phones as a platform, further studies should examine methods of increasing ownership, bridging the digital divide, and empowering communities. Potential studies could include geographic information systems that map gaps in mobile phone penetration rates. In doing so, access to mobile phones can be extended through cost-effective mobile networks such as wireless local loops in remote areas of the country. Improving rural teledensity in developing countries while increasing levels of purchasing power is essential to meeting high levels of demand in resource-constrained areas [11]. In addition to income, other demographic factors as outlined in this study could also facilitate the adoption of more recent generation mobile phones that offer more opportunities for connectivity through Internet use and operating capabilities that give rise to an analysis of second-level digital divides [26]. Other studies can assess the usability of mobile phones in specific occupations to investigate potential means toward increasing mobile capacity [27]. Accordingly, policy changes that stimulate economic productivity through more effective usage of mobile telephony could be adopted. Future studies could also incorporate trends in airtime and equipment costs, payment schemes, and competitive pricing as covariates in changing the dynamics of household access to mobile phones.

When conducting further studies, the limitations in this study must be taken into consideration. For instance, parity was negatively associated with ownership in both unadjusted and adjusted assessments suggesting that the odds of owning a mobile phone decrease with every child. However, parity is also inversely proportional to age—multiparous women are likely to be older—indicating evidence of confounding. Husband’s employment was not statistically significant across all years because the sample size for husbands without employment was low (fiscal year 1: n=23; fiscal year 2: n=7; fiscal year 3: n=5) which also happens to be the reference group. This could be evidence of respondent bias as wives reported on the behalf of their husbands. These limitations must be accounted for to strengthen further analyses.

### Conclusions

This analysis provides insights into the dynamic characteristics of mobile phone ownership over time unveiling the relative contributors to the digital divide; the determinants which initially drive ownership as a privilege of the wealthy gradually lose importance as market and socioeconomic forces increase access even among the poorest members of the population. Still, this “democratization” remains a gradual process and stopgap measures must be pursued to ensure that those living at the very “base of the pyramid” are not further disenfranchised due to their relatively slower uptake of mobile telephony. mHealth and other mobile-facilitated social services targeting the ultra-poor must consider access (or the lack thereof) as an important component of program reach and impact.

The inequitable distribution of power due to differential access to mobile phones underscores the importance of reconciling the demographic barriers to ownership [11]. Mobile phone adoption rates in vulnerable populations such as households with low education, no electricity, and of low economic standing are of particular interest because they are often the slowest and the last to obtain access, as shown in this analysis. Fortunately, as competition increases and costs of ownership are driven down, affordability increases, especially among lower SES populations [11]. Social innovations, such as Bangladesh’s Village Phone Program, show how communal access to phones can bridge the household mobile phone ownership gap in the lowest SES strata while these groups gradually climb the exponential curve to saturation [3,28]. These data from Bangladesh provide a heartening glimpse into the natural trajectories of the mobile phone revolution, across a sociodemographically heterogeneous population, illustrating how even in developing markets, large gaps in mobile phone ownership are unlikely to persist forever through a combination of natural market forces and technologic/socioeconomic innovation.

## Abbreviations

GSMA: Groupe Speciale Mobile Association
ITU: International Telecommunications Union
RCT: randomized controlled trial
SES: socioeconomic status
UNDESA: United Nations Department of Economic and Social Affairs
UNESCAP: United Nations Economic and Social Commission for Asia and the Pacific
VIF: variation inflation factor
WI: wealth index
